# Total scattering and pair distribution function analysis in modelling disorder in PZN (PbZn_1/3_Nb_2/3_O_3_)

**DOI:** 10.1107/S2052252515018722

**Published:** 2016-01-01

**Authors:** Ross E. Whitfield, Darren J. Goossens, T. Richard Welberry

**Affiliations:** a Research School of Chemistry, Australian National University, Canberra, ACT 0200, Australia; b Neutron Data Analysis and Visualization, Oak Ridge National Laboratory, Oak Ridge, TN 37831, USA; c School of Physical, Environmental and Mathematical Sciences, University of New South Wales, Canberra, ACT 2600, Australia

**Keywords:** total scattering, pair distribution function analysis, modelling disorder, PZN, single-crystal diffuse scattering

## Abstract

The ability of the pair distribution function (PDF) analysis of total scattering (TS) from a powder to determine the local ordering in ferroelectric PZN (PbZn_1/3_Nb_2/3_O_3_) has been explored by comparison with a model established using single-crystal diffuse scattering (SCDS). While X-ray PDF analysis is discussed, the focus is on neutron diffraction results because of the greater extent of the data and the sensitivity of the neutron to oxygen atoms, the behaviour of which is important in PZN. The PDF was shown to be sensitive to many effects not apparent in the average crystal structure, including variations in the *B*-site—O separation distances and the fact that 〈110〉 Pb^2+^ displacements are most likely. A qualitative comparison between SCDS and the PDF shows that some features apparent in SCDS were not apparent in the PDF. These tended to pertain to short-range correlations in the structure, rather than to interatomic separations. For example, in SCDS the short-range alternation of the *B*-site cations was quite apparent in diffuse scattering at (½ ½ ½), whereas it was not apparent in the PDF.

## Abstract 2

The ability of the pair distribution function analysis of total scattering from a powder to determine the local ordering in ferroelectric PZN (PbZn_1/3_Nb_2/3_O_3_) is explored by comparing it with a model established using single-crystal diffuse scattering.

## 1. Introduction   

**Figure 1:**
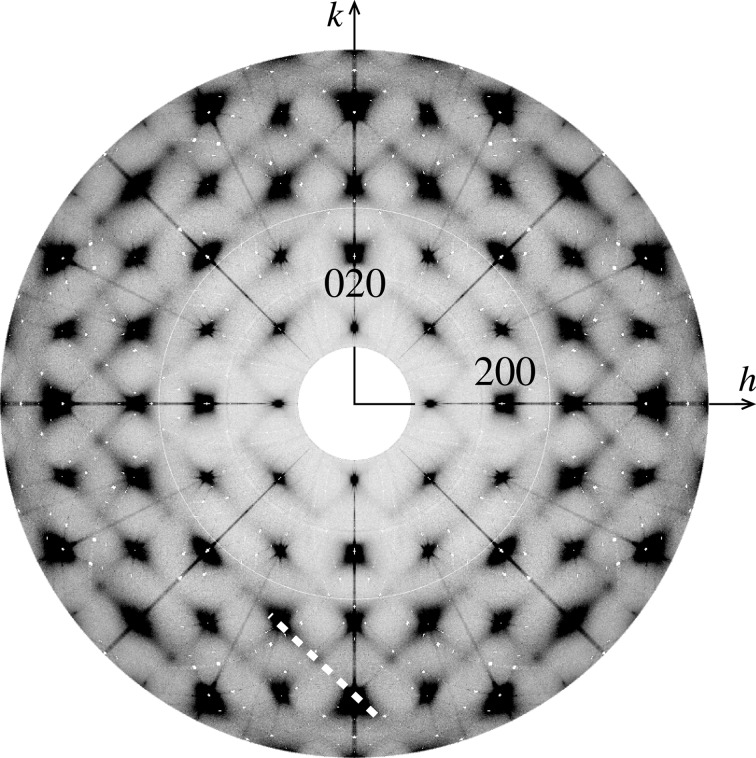
The *hk*0 cut of reciprocal space measured with neutrons at 300 K, with black indicating low intensity and white high. The diagonal white dashed line through (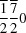) indicates a 〈110〉 rod of scattering.

The existence of local order in ferroelectrics is well established (Honjo *et al.*, 1964 ▸; Randall *et al.*, 1987 ▸). The unequivocal elucidation of this order is less clear, with a range of interpretations available (Bosak & Chernyshov, 2008 ▸; Chernyshov *et al.*, 2011 ▸; Burkovsky *et al.*, 2012 ▸; Bokov & Ye, 2006 ▸; Welberry *et al.*, 2006 ▸; Welberry *et al.*, 2005 ▸). Of the relaxor ferroelectrics, PZN (PbZn_1/3_Nb_2/3_O_3_) is of great interest, partly because of its technological significance, and partly because it and many similar and derived compounds are available not only as powders but as single crystals. This allows highly detailed studies of the local ordering to be undertaken, using single-crystal diffuse scattering (SCDS). The *hk*0 cut of reciprocal space for PZN is shown in Fig. 1 ▸ (Whitfield *et al.*, 2012 ▸). SCDS data can be collected across three-dimensional volumes of reciprocal space. This makes analysis of SCDS a very powerful technique, as it is sensitive to features which occupy only small regions of reciprocal space yet are very descriptive of the local ordering, and it is also sensitive to highly anisotropic features. If only a powder or polycrystal of a material is available, the data are by necessity averaged such that only a one-dimensional powder diffraction pattern can be obtained, possibly buttressed with electron diffraction from a single grain; there are many examples of this (Withers *et al.*, 2003 ▸).

If the powder diffraction pattern is obtained with care, and can be rigorously normalized such that the normalized scattering function *S*(*Q*) can be obtained, then these data can be transformed to give the pair distribution function (PDF), which is able to give insight into local ordering in materials (Proffen & Billinge, 1999 ▸; Proffen *et al.*, 2002 ▸; Neder & Proffen, 2009 ▸; Billinge & Kanatzidis, 2004 ▸; Billinge & Thorpe, 2002 ▸).

Mathematically, if the scattering is described by the function *S*(*Q*), then
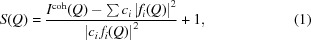
where *I* ^coh^ is the observed intensity at *Q, c_i_* are the atomic concentrations and *f_i_* are the X-ray form factors. The pair distribution function (PDF), *G*(*r*), is then calculated using
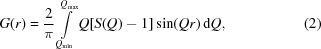
where the choice of *Q*_min_ and *Q*_max_ has an influence on the calculated *G*(*r*) (Proffen & Billinge, 1999 ▸).

Note that equation (2) is not restricted to the Bragg positions – it uses *all* of *S*(*Q*). This means it looks at the ‘total scattering’ (TS) from the sample, which, compared with SCDS, has the advantage of being easier and quicker to collect, while the PDF analysis allows more straightforward refinement of the data. Hence, the PDF has become extremely popular and widely used: it allows the study of samples that cannot be grown as crystals, it requires less specialist knowledge than analysis of SCDS, it can be more rapidly collected as a function of temperature and applied field, and so on. It is therefore interesting to apply it to a material for which high-quality SCDS data have been obtained and modelled, to explore the sensitivity of the PDF approach to various structures and orderings in the sample, and to explore the degree to which it can be reliably interpreted.

There are many tools that can be used to undertake PDF analysis, and these can be used in many different ways and to many levels of complexity. The work here focuses on relatively elementary use of the software *PDFgui* (Farrow *et al.*, 2007 ▸) and on the use of reverse Monte Carlo (RMC) techniques using the software *DISCUS* (Proffen & Neder, 1997 ▸; Neder & Proffen, 2009 ▸). This is because not all possibilities can be undertaken, and because the focus is on the sensitivity of the PDF to various classes of features. Further variations can always be explored in the future.

### 1.1. Data collection and processing   

Data for PZN were collected in the temperature range 100–500 K using both X-rays and neutrons. The X-ray data were collected using beamline 11-ID-B at the Advanced Photon Source (APS; Argonne National Laboratory, Illinois, USA) with data collected every 2.5 K from 100 up to 500 K with a total of 161 data sets reduced using *PDFgetX2* (Qiu *et al.*, 2004 ▸). The neutron data were collected using the NPDF beamline (Proffen *et al.*, 2002 ▸) at the Lujan Neutron Scattering Center (Los Alamos National Laboratory, New Mexico, USA) with 15 data sets collected at temperatures ranging from 100 to 500 K reduced using *PDFgetN* (Peterson *et al.*, 2000 ▸).

At NPDF, samples were measured in standard vanadium cans (radius 2.945 mm) in a top-loading Displex cryostat at a range of temperatures. Measurements were collected every 25 K, starting at 100 K and heating up to 500 K, with a collection time of 3 h per temperature. A longer collection time of 8 h was used for the room-temperature measurement to allow a higher quality PDF to be produced and also to help evaluate the statistics of the shorter 3 h runs.

At APS, the wavelength was 0.2128 Å (58.2636 keV), the beam size was 1 × 1 mm and the detector used was a Perkin–Elmer amorphous silicon detector. The sample environment was an Oxford Cryosystems Cryostream 700+, with the sample placed in a 1 mm diameter Kapton tube. The configuration was a multi-purpose one, being used for a range of experiments, including SCDS, and was perhaps not optimal for a PDF experiment. As a result, the work presented here focuses more heavily on the neutron data.

The X-ray data were processed with a *Q*_max_ of 26 Å^−1^, while the neutron data used a *Q*_max_ of 40 Å^−1^. The data were processed to produce PDFs out to maximum *r* values of 20 and 80 Å for X-rays and neutrons, respectively.

**Figure 2:**
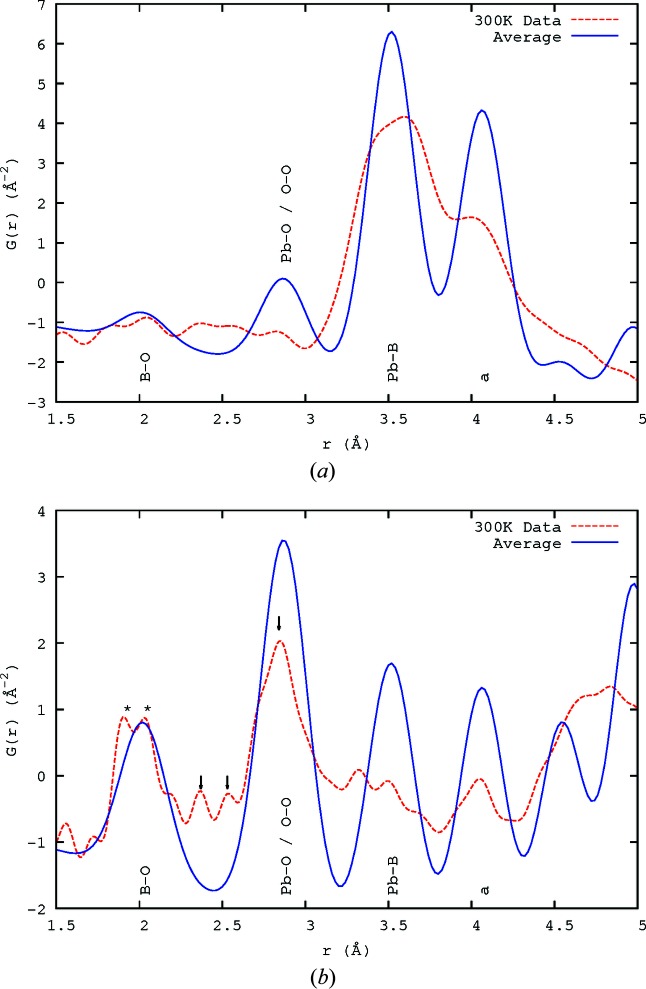
The calculated PDF from the average structure at 300 K (Forrester *et al.*, 2006 ▸; Kisi *et al.*, 2006 ▸; Terado *et al.*, 2006 ▸) compared with the 300 K PDF data obtained using (*a*) X-rays and (*b*) neutrons. The label ‘a’ indicates the lattice *a* parameter, which includes Pb—Pb, *B*-site—*B*-site and O—O atom pairs. Asterisks * indicate the *B*-site—O peaks (denoted B—O) in the neutron data in part (*b*). The arrows in part (*b*) show the peaks corresponding to the different Pb—O distances that result from displacement of Pb^2+^ towards the nearest O atoms.

The difference between the PDF calculated from the average structure of PZN (Forrester *et al.*, 2006 ▸; Kisi *et al.*, 2006 ▸; Terado *et al.*, 2006 ▸) and the data collected at 300 K is shown in Fig. 2 ▸ for both X-ray and neutron data up to 5 Å. The observations show many split peaks (an example is starred in Fig. 2 ▸ *b*), indicating that the interatomic distances apparent in the average structure are really averages over multi-modal distributions.

## 2. Initial interpretation   

From bond-valence calculations (Brese & O’Keeffe, 1991 ▸), the preferred separations of Zn and Nb from the nearest O atoms are 2.11 and 1.97 Å, respectively. In Fig. 2 ▸(*b*), the *B*-site—O peak (denoted *) is split into two, with the maxima occurring at 1.91 and 2.03 Å, relatively close to what might be expected. This splitting of the peak was confirmed to be real (not an artefact of the PDF transform) by varying the *Q*_max_ of the transform and comparing the results.

The peak at the distance corresponding to the lattice parameter *a* (shown in Figs. 2 ▸ *a* and 2 ▸ *b*) includes the atom pairs Pb—Pb, *B*-site—*B*-site and O—O. That the intensity of this peak is significantly less than that expected from the average structure for both X-rays and neutrons implies that there are relatively few pairs of atoms which have this separation and that there is a large amount of disorder on the nanoscale.

**Figure 3:**
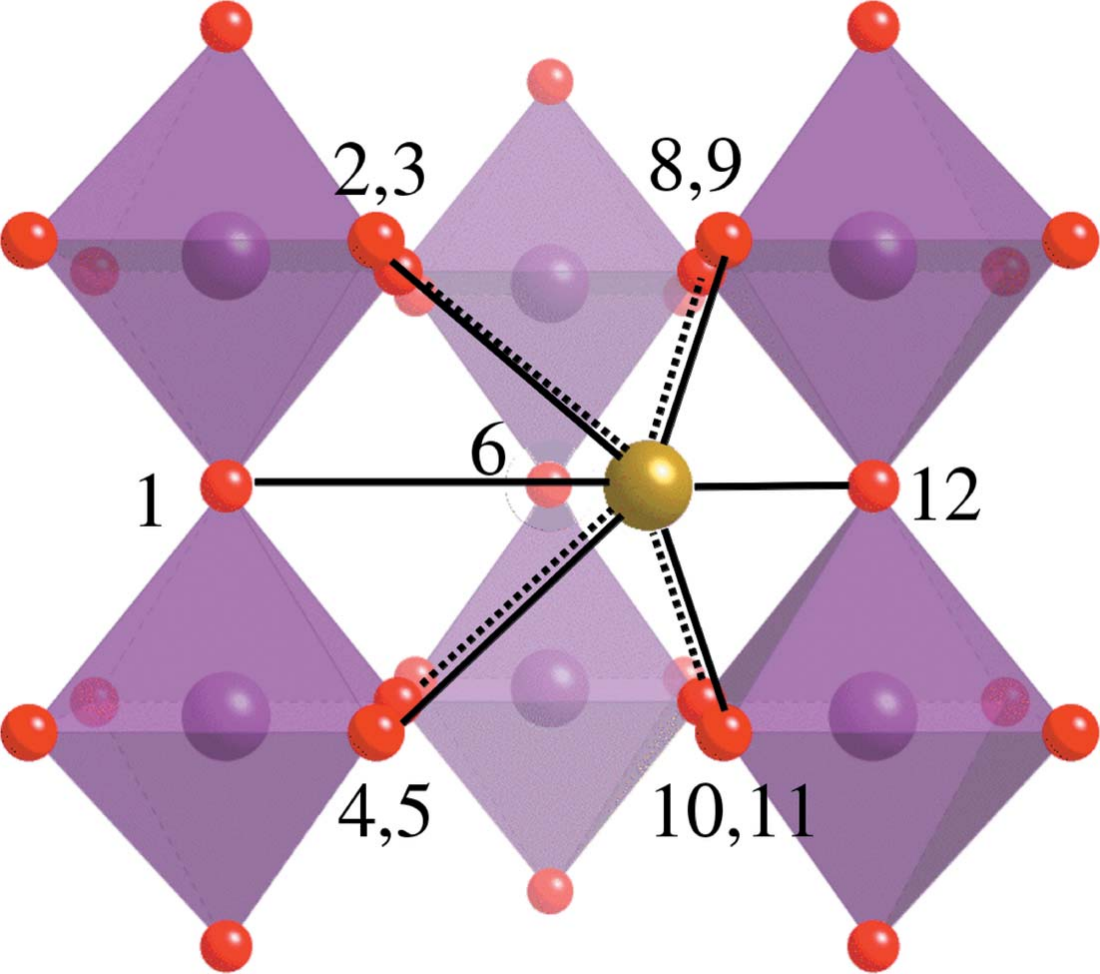
A schematic diagram of a Pb^2+^ ion shifting along the 〈110〉 direction. The motion is towards O12, away from O1 and largely perpendicular to the remaining atoms (O7 is out of the plane of the picture).

Looking more closely, the Pb—O peak(s) (arrowed in Fig. 2 ▸ *b*) show that there is no single Pb—O distance present, rather a set of distances, mostly clustered around the average (the large peak at ∼2.84 Å is clearly asymmetric) but with other distances present – shorter peaks occur at 2.38 and 2.54 Å. There is a larger *r* peak occurring at around 3.32 Å which is also due to a Pb—O separation. The large peak at 2.84 Å corresponds to the distance to the O atoms perpendicular to the displacement direction; its large size suggests that this is the majority of the oxygen ions in the Pb environment. That (2.38 + 3.32)/2 = 2.85 ≃ 2.84 strongly suggests that a given Pb atom is displaced towards one O atom and away from another. This would tie in well with the model based on 〈110〉 displacements (Welberry *et al.*, 2005 ▸), in which the Pb atom is displaced towards one of the O atoms in its coordination shell and the motion is largely perpendicular to the remaining O atoms. Fig. 3 ▸ attempts to illustrate this. The Pb^2+^ ion moves towards O12, away from O1, shortens its distance to O8 and O11 marginally, and lengthens the remaining distances to varying degrees – agreeing with the major Pb—O peak being asymmetric and higher at longer *r*. It is not clear that this explains the origin of the 2.54 Å peak, but then it is likely that the real displacement structure is more complex than this simple picture. Nevertheless, while it may be possible to model the SCDS without assuming 〈110〉 displacements (Bosak & Chernyshov, 2008 ▸; Hlinka, 2012 ▸; Paściak & Welberry, 2011 ▸; Paściak *et al.*, 2007 ▸; Paściak *et al.*, 2012 ▸; Goossens, 2013 ▸), these observed absolute distances tie in well with this as being the real displacement direction in the material.

**Figure 4:**
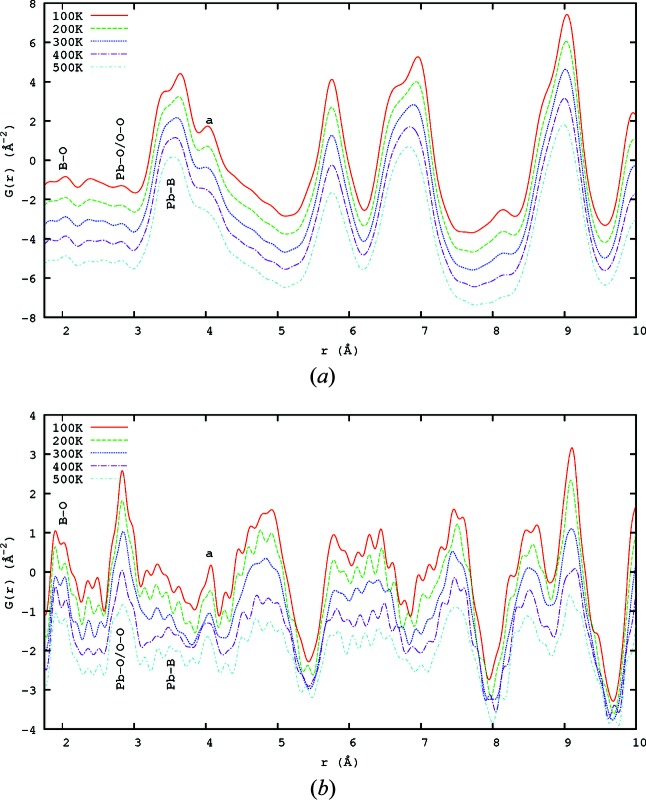
The evolution of (*a*) the X-ray and (*b*) the neutron PDFs from 100 to 500 K, shown every 100 K, 1.75 < *r* < 10 Å. The PDF evolves slowly across the phase transition at around 400 K. Elements are indicated by their chemical symbols, except that B denotes a *B*-site element (Zn or Nb).

A plot showing the *r* < 10 Å range of the X-ray and neutron PDFs from 100 to 500 K is shown in Fig. 4 ▸. There is a small shift to higher *r* of the *B*-site—O peaks (at around 2 Å), which is consonant with the variation in lattice parameter. For the other peaks, a decrease in temperature brings an increase in the peak sharpness. With higher temperature there is an increase in the thermal motion of the atoms which broadens the distribution of atomic separations. Because the relaxor phase transition is broad and the domain structure persists through it, there is no strong signature of the transition in the scattering, just as there is none in SCDS, except possibly the temperature dependence of the atomic size-effect (Whitfield *et al.*, 2012 ▸).

It is apparent that the sensitivity of neutrons to oxygen greatly increases the information available relative to the X-ray patterns.

## 3. PDF calculation from a model based on SCDS   

To explore the sensitivity of the PDF to the forms of disorder believed to occur in PZN, neutron PDFs were calculated at each different stage in the Monte Carlo (MC) simulation process used to implement an SCDS model (Welberry *et al.*, 2006 ▸). Hence, this model was not being fitted to the PDF; rather, the PDF was being compared with predictions from the model.

The model consisted of an array of 50 × 50 × 50 unit cells. By putting potentials (often relatively artificial ones) on the atoms in the model and allowing the model to relax through MC simulation, local correlations were introduced. Fourier transform of the model then allowed comparison of the model diffraction pattern with the observed SCDS (Barabash *et al.*, 2009 ▸; Welberry, 2004 ▸). Similarly, the PDF of the model was calculated and compared with observations.

The key steps that were found necessary to model the SCDS data from PZN were as follows (Welberry *et al.*, 2006 ▸; Whitfield, 2013 ▸; Whitfield *et al.*, 2014 ▸):

(1) Populate a model crystal according to the average structure. A cubic structure with *a* = 4.06 Å was used.

(2) Negatively correlate the occupancies of the *B*-sites (Nb and Zn) such that they try to alternate. This cannot result in long-range order because Nb and Zn do not occur in the ratio 1:1.

(3) Randomly displace each Pb ion in one of the 12 〈110〉 directions by an amount γ_Pb_ ≃ 0.307 Å.

(4) Use an MC simulation to cause these displacements to become correlated and aggregate into planar domains normal to the 〈110〉 directions. The domains created vary in size up to around ten unit cells or about 40 Å, as shown by Whitfield *et al.* (2014 ▸).

(5) Implement an atomic size-effect to adjust the relative separation of adjacent Pb ions depending on the relative orientations of their displacements. This step increases the Pb—Pb displacement correlation from 0.142 to 0.562.

(6) Position the non-Pb atoms into the structure in a way which is consistent with the average structure and minimizes the global instability index (Lufaso & Woodward, 2001 ▸).

It is then possible to explore not just whether the PDF and the model of SCDS agree – useful in itself, as the model crystal can then be tested against a different experiment – but also whether the PDF is sensitive to the changes that occur in the model after each step. The SCDS changes noticeably at each step (Welberry *et al.*, 2006 ▸, 2005 ▸; Whitfield *et al.*, 2014 ▸). Indeed, the different aspects of the model were added *because* the SCDS could not be adequately modelled without them, and therefore each has a clear signal in the SCDS.

**Figure 5:**
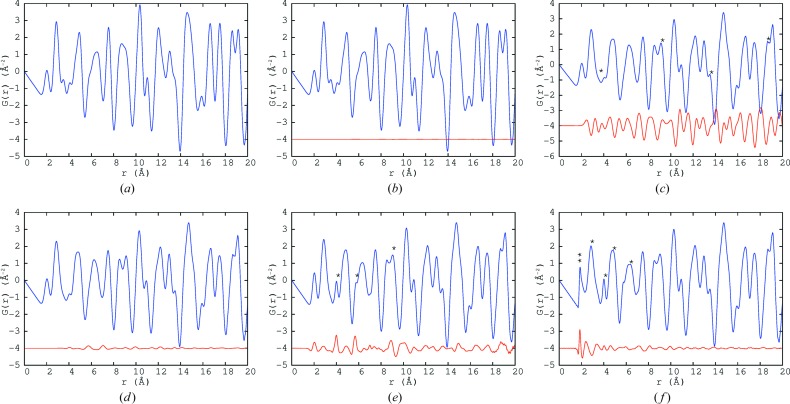
The neutron PDF calculated (blue lines) at different stages in the development of the SCDS model. The difference between the current and previous step calculated is shown as a red line in each case. (*a*) Average PZN. (*b*) After *B*-site Ising model. (*c*) Pb 〈110〉 displacement. (*d*) Nanodomains formed. (*e*) Size-effect applied. (*f*) BVS MC. Positions labelled with * are discussed in the text.

The PDFs at six different steps of the MC process were calculated out to 100 Å, the plots of which (showing the *r* range up to 20 Å) are given in Fig. 5 ▸. The first step (Fig. 5 ▸ *a*) is the average initial starting structure of the PZN crystal with only atomic displacement parameters (ADPs), taken from the literature (Terado *et al.*, 2006 ▸), applied. For all the PDF calculations, the resolution damping and broadening parameters from NPDF were used, 0.00623 and 0.0021, respectively, as provided by the instrument scientist.

The next step (Fig. 5 ▸ *b*) is after the Ising model has been applied, causing the *B*-site chemical ordering of Zn and Nb, as shown by Whitfield *et al.* (2014 ▸). This has no apparent effect on the PDF, though because Nb and Zn have different chemistry it may manifest itself after displacement correlations have been applied, particularly in the first *B*-site—O peak at around 2 Å. The effect of the *B*-site chemical ordering on the SCDS was the formation of diffuse peaks occurring in the (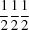) type positions and was readily apparent.

In Fig. 5 ▸(*c*), the Pb displacements were applied randomly such that each Pb was displaced in one of the 12 〈110〉 directions, while keeping the average ADPs the same. This had a big impact on the splitting of some peaks, highlighted with *, particularly at 9 and 19 Å. It also caused most of the peaks to broaden or change in shape.

*Via* MC simulation, the Pb displacements were then formed into planar nanodomains, as shown in Whitfield *et al.* (2014 ▸). This produced the rods of diffuse scattering seen in the SCDS (Fig. 1 ▸). In the resulting PDF (Fig. 5 ▸ *d*) there was only a small change in the sharpness of some of the peaks. Thus, it would appear that the PDF is not very sensitive to the formation of planar nanodomains.

The size-effect serves to prefer some interatomic Pb—Pb distances, depending on the mutual orientations of the displacements of the two Pb ions, without altering the planar domain distribution. This is seen in Fig. 5 ▸(*e*) as an increase in the intensity and sharpness of some peaks, particularly those marked with *. This includes the peak at the unit-cell length, around 4 Å, which is the distance between neighbouring Pb atoms.

When the bond-valence sum (BVS) based MC energy was applied to position the non-Pb atoms (Whitfield *et al.*, 2014 ▸), strong correlations between the *B*-site and O-atom displacements were formed, which caused the peak at around 2 Å (marked with ** in Fig. 5 ▸ *f*) to sharpen. There were changes in many of the other peaks as well, in particular those highlighted with *.

The difference plot (lower red line in Fig. 5 ▸ *f*) shows that the MC model using the BVS-based energy term had its greatest effect when *r* < 4 Å, a length scale dominated by nearest-neighbour interactions.

**Figure 6:**
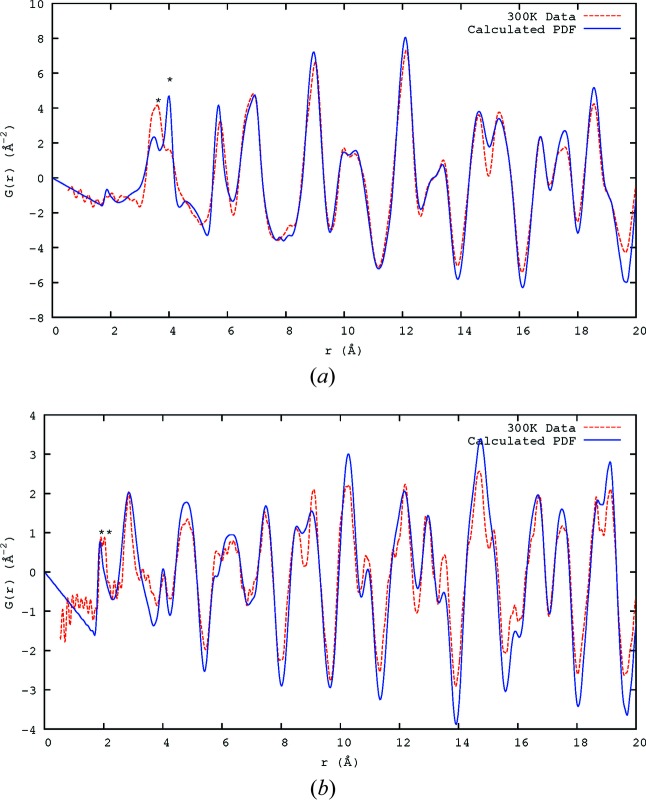
Plots of the PDF calculated from the SCDS model (blue lines) compared with the X-ray and neutron PDF data collected at 300 K (dashed red lines). (*a*) X-ray PDF, observed and calculated from SCDS model. (*b*) Neutron PDF, observed and calculated from SCDS model.

When comparing the final model with the 300 K X-ray and neutron data (Fig. 6 ▸), it can be seen that, while there is good agreement between the single-crystal model and the PDF, there are some major differences. The most obvious in the X-ray data is the difference in the peaks at around 4 Å, marked with * in Fig. 6 ▸(*a*). This is the peak corresponding to the unit-cell length, *a*, with the major contributor being the Pb—Pb pairs. This shows that the two different distances, 3.45 and 4.00 Å, seem to be consistent between the observed PDF and that calculated from the SCDS model, but the number of pairs with each bond length is not. This shows that the simulation does create the interatomic spacings but not the displacement populations observed in the experimental PDF. The neutron single-crystal model PDF is generally broader than the observed PDF, though again the *r* values of the peaks agree well.

It should be noted that some features are inherently more suited to exploration using PDF or SCDS. For example, a reciprocal space view (SCDS) is ideal for observing the *B*-site ordering, since the scattering from this coalesces at well defined reciprocal space points. The PDF is useful for local bonding distances, since these are localized in real space. This is one reason why the refinement of a structural model against the PDF and the TS data – regardless of whether SCDS is relevant to the problem at hand – comes highly recommended. Unfortunately, it is not possible in one publication to consider all possible combinations of approaches to data analysis, so this analysis is not dealt with here.

The SCDS model was developed qualitatively, while PDF data are quite quantitative. Thus, it is reasonable that we have a qualitative agreement about the existence or not of peaks, but not a quantitative agreement regarding their heights. The concordance is good when it is recalled that this is not a fit but a prediction. And, regardless, some features that cause changes in the calculated SCDS (and agree with the observed SCDS) do not change the calculated PDF, suggesting that the SCDS contains information that cannot be seen in the PDF, and that the quantitative information that is perhaps more readily available from the PDF could be used to improve the modelling of the SCDS, by contributing to defining the population of interatomic separations that are correlated by the MC model.

## 4. *PDFgui* analysis   

*PDFgui* (Farrow *et al.*, 2007 ▸) is a program for full-profile fitting of PDF data using a Rietveld-style refinement. The program allows the sequential fitting of multiple sets of PDF data so that the evolution of the structure with temperature can be seen. The fit was done using an initial model based on the structure found by Kisi *et al.* (2006 ▸) using Rietveld refinement of powder patterns. The starting model has the *R*3*m* space group with the Pb atom at 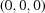, the Nb/Zn *B*-site at (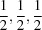) and the O atoms at the (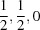) positions. The best fit to the PDF was a rhombohedral structure at low temperature and a cubic one at high temperature. The limitation of this type of modelling is the inability to introduce the displacement correlations that are expected. The model cannot contain *B*-site ordering.

To better model the short-range ordering on the length scale expected, including the planar domain formation, reverse MC (RMC) modelling was used, and that is explored in §5. However, *PDFgui* provided a preliminary study of the data and gave many insights.

### 4.1. X-ray data   

**Figure 7:**
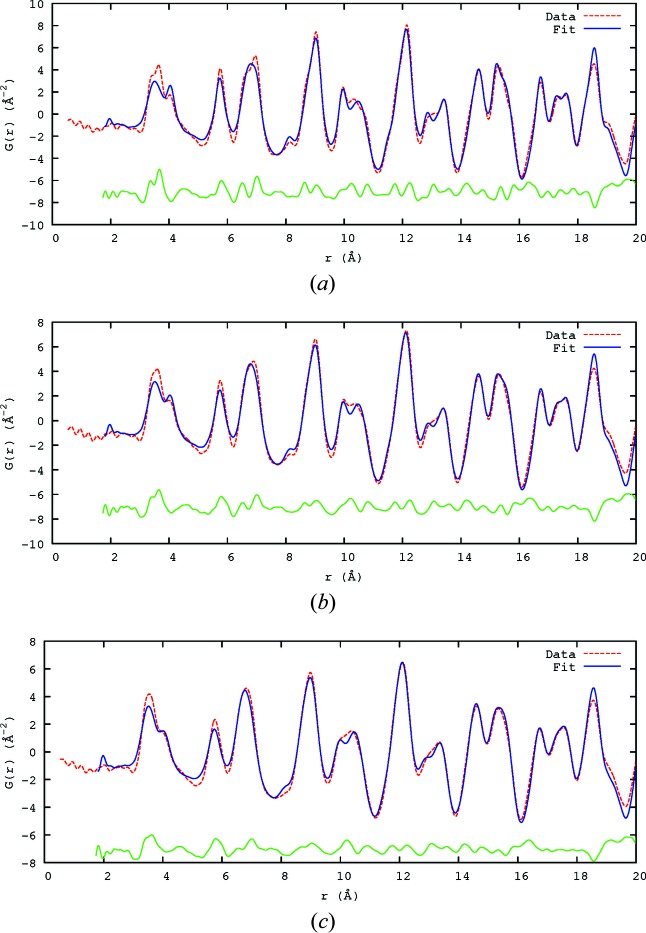
The fitted 100, 300 and 500 K X-ray PDF data. Shown are the observed trace (dashed red lines), the calculated fit using *PDFgui* (blue lines) and the difference (green lines), offset to allow easier viewing. (*a*) 100 K, (*b*) 300 K, (*c*) 500 K.

The X-ray PDF data have the limitation of being collected out to *Q*_max_ = 26 Å. This limits the resolution and the quality of the resulting PDF. *G*(*r*) was calculated to the limit of *r* = 20 Å. The best fits to the data are shown in Fig. 7 ▸ for the temperatures 100, 300 and 500 K. The fits are better at higher *r* because the local order most strongly affects the small-*r* features, and an average structure model cannot take local order into account. It *is* possible to perform multiple *PDFgui* refinements over different distance ranges and to infer details of structure from changes, but the fact remains that a single unit cell cannot model effects that extend for multiple cells.

**Figure 8:**
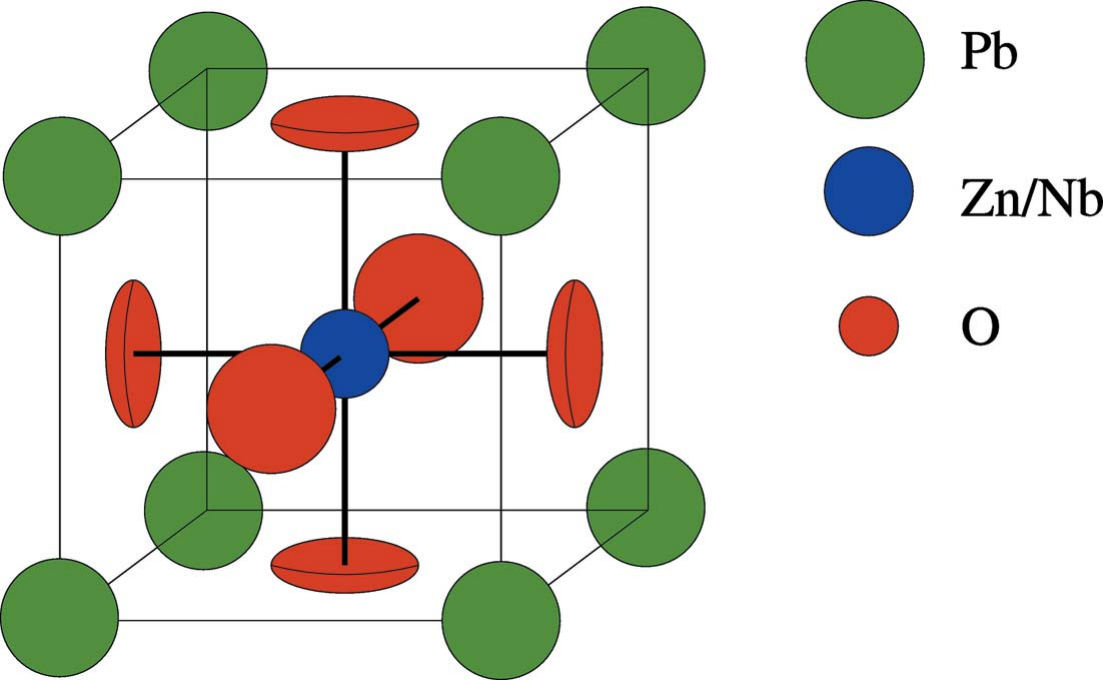
The PZN unit cell, showing the oblate ellipsoidal shape of the distribution of oxygen. The O atoms have an anisotropic broadening that is larger in the plane of the nearest four Pb atoms.

The fit used isotropic ADPs, except in the case of the O atoms, where anisotropic ADPs allowed different parameters for in- and out-of-plane directions, as shown in Fig. 8 ▸. There was also large uncertainty on the rhombohedral angle and the angle was fixed. The ADPs found for the fits of the 100, 300 and 500 K data are shown in Table 1 ▸. The large ADP for Pb is indicative of the ionic displacements. These are still in the ranges given by Forrester *et al.* (2006 ▸) and Kisi *et al.* (2006 ▸), with the exception of the large ADPs for O, which are due to the insensitivity of X-rays to O compared with the other elements present.

### 4.2. Neutron data   

**Figure 9:**
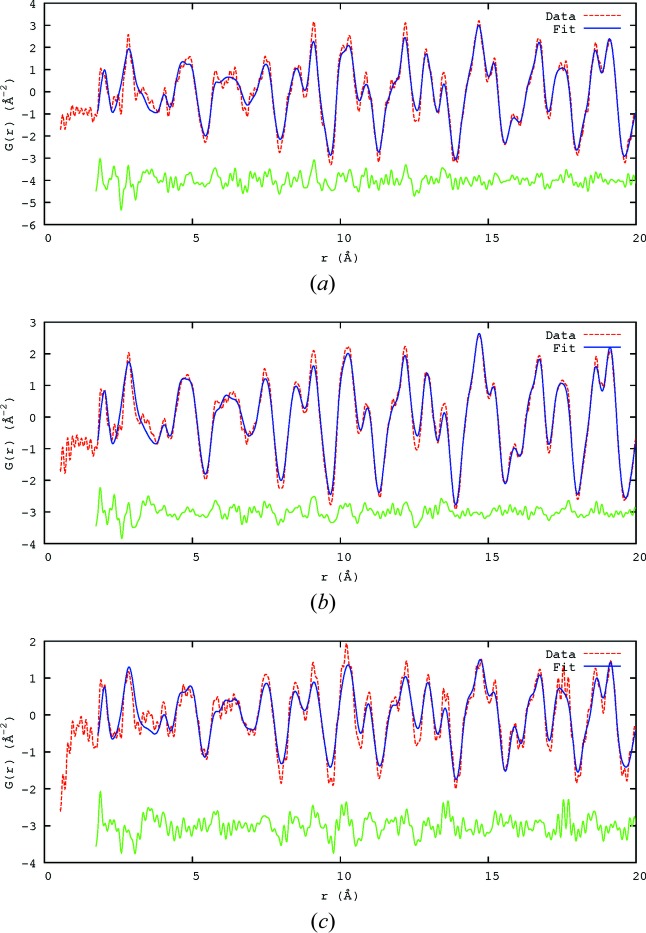
The fitted 100, 300 and 500 K neutron data, fitted out to 80 Å using *PDFgui*, shown out to 20 Å. Shown are the data, the calculated fit and the difference, using the same colour scheme as in Fig. 7 ▸, offset to allow easier viewing. (*a*) 100 K, (*b*) 300 K, (*c*) 500 K

The neutron data were fitted in the range 1.75–80.0 Å. The best fits to the data are shown in Table 2 ▸ for 100, 300 and 500 K. The fits show reasonable agreement (Fig. 9 ▸); the disagreement is greatest for the range *r* < 10 Å, giving strong and direct evidence of the need to allow for short-range order (SRO), something not directly apparent from conventional Rietveld fitting of the powder diffraction profile, where the disorder must be inferred from the large Pb anisotropic ADPs.

**Figure 10:**
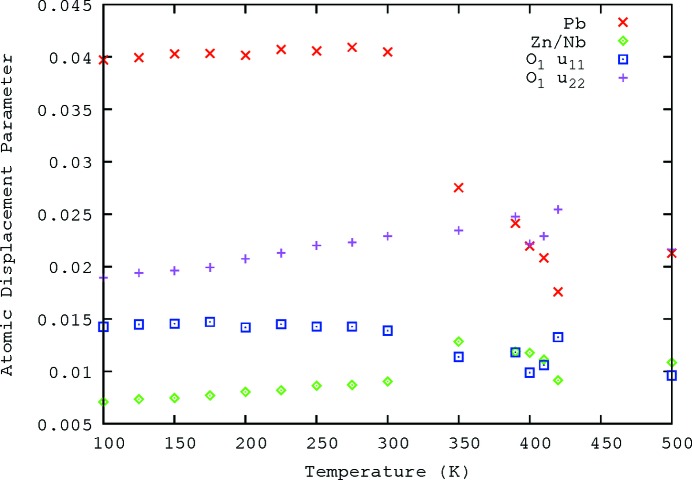
Trends in the ADPs of the atoms in PZN, from fitting the neutron PDF, as a function of temperature, *T*.

The ADPs vary differently for the different atoms, as shown in Fig. 10 ▸. The Pb atoms show a *decrease* in ADPs, with a dramatic change close to the phase transition, in contrast with what is seen with the X-ray scattering. This is important information, as it suggests that the PDF should be able to help quantitatively determine δ_Pb_ (to feed into SCDS fitting, for example). The other atoms, Zn, Nb and O, change in a similar fashion to that seen in the X-ray PDF data (Table 1 ▸).

In summary, the analysis of the X-ray PDF data using *PDFgui* offers a very useful intermediate step between conventional powder diffraction analysis (Rietveld) and fuller analysis of PDF (*e.g.* RMC) and SCDS.

The results found here compare well with what has been found previously. The patterns of evolution of the rhombohedral angle and lattice parameter are similar to those observed in powder diffraction analysis (Forrester *et al.*, 2006 ▸; Kisi *et al.*, 2006 ▸).

For modelling SRO, a larger model using a more powerful modelling technique was needed. Hence, RMC modelling is explored in the next section. The results from the *PDFgui* analysis were used as a starting model for the RMC modelling.

## 5. RMC modelling   

RMC modelling (Keen, 1998 ▸; McGreevy & Pusztai, 1988 ▸; Nield, 1998 ▸) allows the PDF data to be fitted with a large-scale model, allowing exploration of SRO. In the case of PZN, model sizes of 10 × 10 × 10 and 20 × 20 × 20 unit cells were explored. The former was large enough to contain nearest-neighbour interactions and to be statistically significant, while the latter allowed the effects of increasing model size to be examined. Both models were smaller than that used for the SCDS simulations because of the significant time needed for RMC modelling on a large model, and because if the model is too large a single MC step will not have a sufficiently significant effect on the goodness of fit.

In the modelling undertaken here, *B*-site occupancies were changed *via* swapping atoms, and the ADPs of similar atom types were swapped, in both cases preserving the global averages. This was similar to the method used to construct the single-crystal model. The goodness of fit, χ^2^, was used, as in equation (3), to determine if a swap improved the model, with Δχ^2^ calculated using equation (4) (Proffen & Neder, 1999 ▸; Neder & Proffen, 2009 ▸).
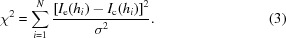
Equation (3) (Proffen & Neder, 1999 ▸; Neder & Proffen, 2009 ▸) is measured over all data points *h_i_*, and *I*_e_ is the experimental intensity and *I*_c_ the calculated intensity. The σ^2^ in the equation is a scaling parameter independent of the intensity in the calculation.
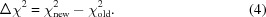
All changes with Δχ^2^ < 0 are accepted. A change with Δχ^2^ > 0 may be accepted with a probability of *P* = exp(−Δχ^2^/2) (Proffen & Neder, 1999 ▸; Neder & Proffen, 2009 ▸). The σ^2^ term affects the probability of the change being accepted. If σ^2^ = 0 then *P* = 0 and only changes that improve the fit, Δχ^2^ < 0, will be accepted.

### 5.1. Initial starting model   

The initial model used for RMC was based on a model determined from *PDFgui* but expanded to multiple unit cells. The basis of the model was the best fit of the 300 K neutron data with a reduced set of parameters (Table 3 ▸), using only isotropic ADPs for compatibility with the RMC modelling program used, *DISCUS* (Proffen & Neder, 1997 ▸; Neder & Proffen, 2009 ▸). The data were fitted using *PDFgui* out to a large *r* range of up to 80 Å, as this provided the best starting model, although the RMC modelling was only refined out to 20 Å. The focus of the RMC fitting was on the 300 K neutron data as this was the highest quality data collected.

The fit quality of the initial starting model was χ^2^ = 0.0691 for a fit range of 1.75 < *r* < 20.0 Å. The ranges explored in the fitting were 1.75 < *r* < 8 Å and 1.75 < *r* < 20 Å. The two different ranges allowed for the fact that a fit using only low-*r* data might be more sensitive to the nearest-neighbour separations. There are time considerations with fitting the data out to a large range, such as up to 80 Å, where it becomes impractical. The model size, 10 × 10 × 10 unit cells, also limits the refinement range out to ∼40 Å.

The initial parameters used for both small and large models are shown in Table 3 ▸. The model fits well at larger *r*, where short-range effects are less important, and serves well as the starting point to the RMC modelling.

In the initial starting model, there were no correlations between the atomic displacements or the occupancies of the *B*-sites, as the atoms were randomly placed in the model, but with a distribution such that the average across it gave the desired ADPs and composition.

Tests found that a model crystal size of 10 × 10 × 10 unit cells, with a total of 5000 atoms, was large enough to produce a high-quality simulated PDF while still being small enough to be fast to calculate. The size was also large enough to produce local correlations on some of the length scales expected.

The RMC simulation was run over a number of loops with decreasing σ^2^. Each loop consisted of five cycles, where one cycle was equal to the number of iterations though the RMC simulation, equivalent to the total number of atoms in the model (5000). Since during each RMC iteration atoms were swapped, two atoms were visited in each iteration and, on average, they were visited ten times in each loop.

The simulation started with σ^2^ = 0.012 but this was reduced with each of the five loops of five cycles down to σ^2^ = 0 for the last loop. The slow decrease in σ^2^ allowed a better chance of the best fit being found (Neder & Proffen, 2009 ▸).

**Figure 11:**
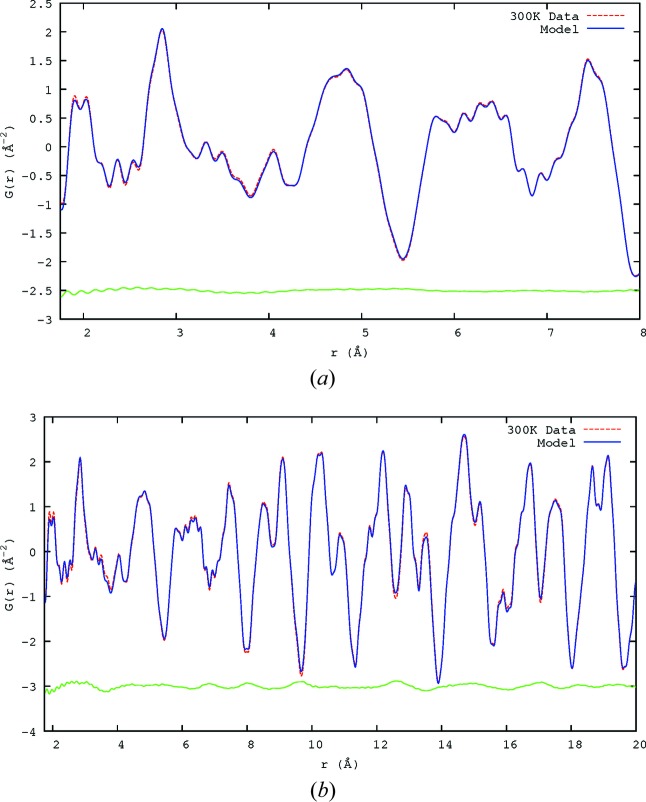
The observed neutron PDFs and those calculated from the RMC model over the two *r* ranges used, (*a*) 1.75 < *r* < 8 Å and (*b*) 1.75 < *r* < 20 Å

Fig. 11 ▸ shows the observed and calculated PDFs over the two ranges used in the fittings. The models show good agreement with the data. For the fit range of 1.75 < *r* < 20.0 Å the final χ^2^ was 0.0026, and χ^2^ = 0.00063 when the fit was restricted to 1.75 < *r* < 8 Å.

### 5.2. Correlations   

Only weak displacement and occupancy correlations were found as a result of the RMC modelling. The strongest correlations which formed, as calculated from equations (5) and (6), are shown in Table 4 ▸ for occupancy correlations of the *B*-site and in Table 5 ▸ for the displacement correlations. 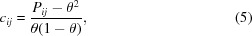


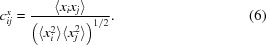
A negative occupancy correlation in the 〈100〉 direction was found to form for the 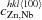 as expected, but it was much weaker than that inferred from the SCDS modelling. This agrees with the earlier qualitative results in §3, which suggested that introducing *B*-site ordering had very little effect on the form of the PDF.

Looking at the displacement correlations in Table 5 ▸, only weak correlations have formed. The strongest of these occurs between the O and *B*-site atoms with a positive correlation in the direction of the vector between the atoms. The displacement correlations for the O and *B*-site in the other directions are very small. Between the Pb and O atoms there is a negative correlation for the directions towards the neighbouring O atom, 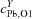 and 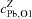, which is the same as was found to be necessary through qualitative arguments during single-crystal modelling (Welberry *et al.*, 2005 ▸, 2006 ▸) and which also arose out of the BVS modelling through crystal chemical considerations (Whitfield *et al.*, 2014 ▸). There was also a positive correlation between the Pb and *B*-site displacements which is also consistent with the single-crystal modelling. Surprisingly, there was no correlation formed for the Pb–Pb atom pairs as would be expected due to the planar nano­domain structure. Equivalent results were found when the model size was increased to 20 × 20 × 20 unit cells.

### 5.3. Calculation of SCDS from the RMC model   

**Figure 12:**
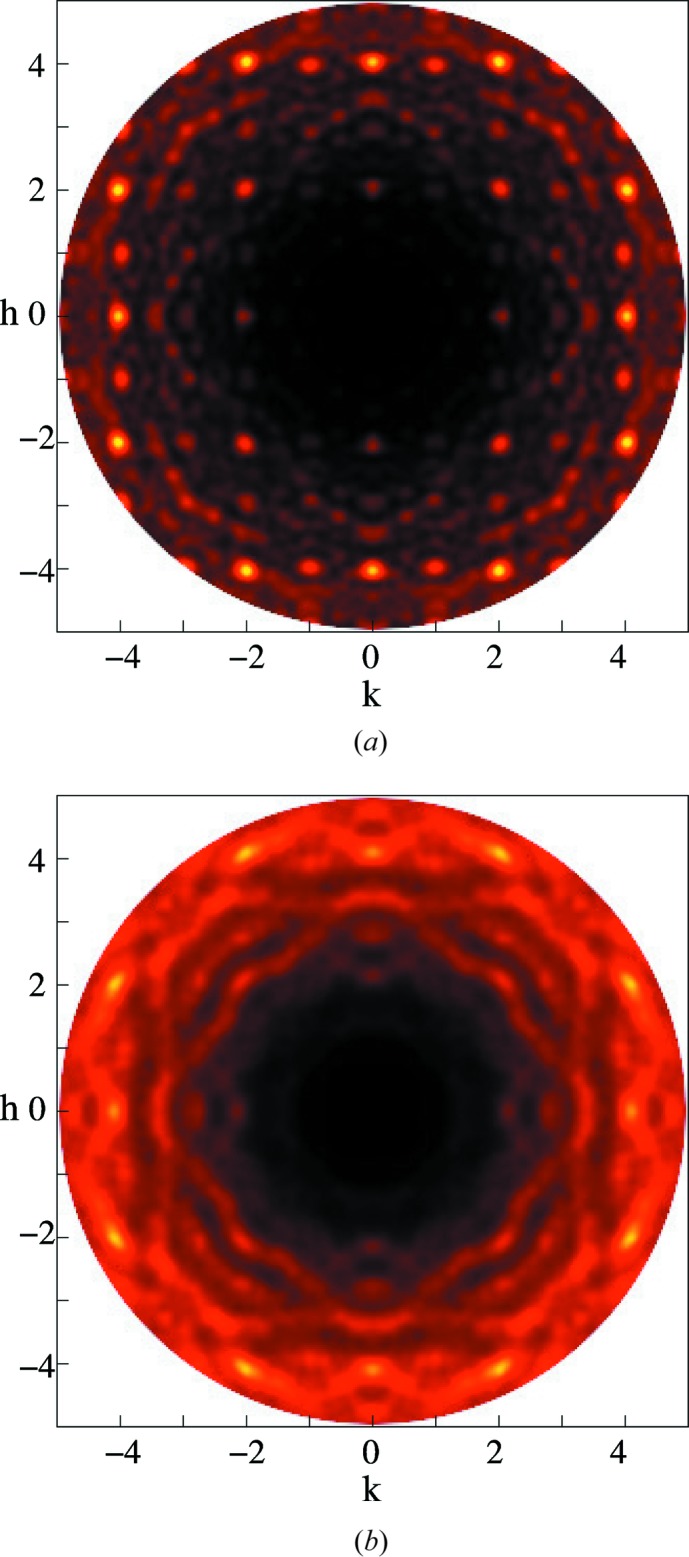
The *hk*0 SCDS plane calculated from two different-sized RMC models that had been fitted to the 300 K neutron total scattering data. (*a*) 10 × 10 × 10 unit cells, range 1.75 < *r* < 20 Å. (*b*) 20 × 20 × 20 unit cells, range 1.75 < *r* < 8 Å.

The SCDS was calculated from the refined models, small and large. Fig. 12 ▸(*a*) shows the *hk*0 plane from the best fit using the rhombohedral structure and 10 × 10 × 10 unit cells. The model was refined over 1.75 < *r* < 20 Å. The SCDS from this model shows some structured diffuse scattering, particularly around some of the Bragg peaks.

An RMC model of 20 × 20 × 20 cells was fitted to the 300 K neutron PDF data. This model was refined over the range 1.75 < *r* < 8 Å. The larger model allows for better statistics when calculating the SCDS. Fig. 12 ▸(*b*) shows the SCDS calculated from this larger model. The formation of structured diffuse scattering is more apparent. In particular, structured diffuse scattering has formed around the (400) peak and there are broad streaks throughout. Given the limitations of using powder data, Fig. 12 ▸(*b*) is quite promising and shows that carefully constrained modelling of a high-quality PDF can give insight into the three-dimensional local ordered structure. Having said that, as would be expected, the SCDS data contain information that in this case was not found during this refinement of the PDF data.

### 5.4. Planar domain formation and *B*-site ordering   

**Figure 13:**
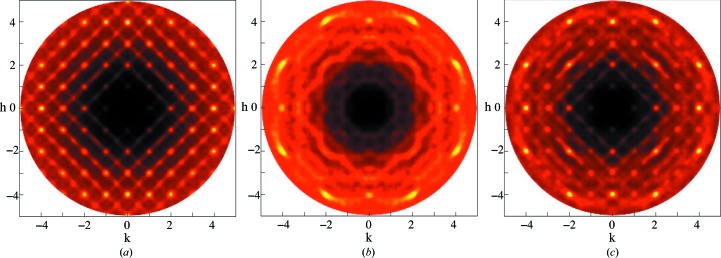
The SCDS calculated from the model crystal resulting from the RMC fit to the PDF of a model in which the starting state already contains polar nanodomains and *B*-site ordering. (*a*) *hk*0 calculated from the starting model. (*b*) and (*c*) *hk*0 calculated from the two different RMC models, where the δ_Pb_ are either allowed to swap or not. (*a*) Starting model with PND and *B*-site ordering. (*b*) After RMC, δ_Pb_ allowed to swap. (*c*) After RMC, δ_Pb_ not allowed to swap.

The planar domains that were used in the SCDS model can be introduced into the RMC model, along with the *B*-site ordering, and the refinement repeated. The larger model size of 20 × 20 × 20 unit cells was used. The starting model contained both the planar nanodomains and the *B*-site chemical ordering, and the diffuse rods can be observed in the SCDS calculated from this in Fig. 13 ▸(*a*).

Two models were explored. In one RMC model the total Pb displacement was allowed to change (‘Swap δ_Pb_’), and so the planar nanodomains were not necessarily conserved. In the other case, only the thermal disorder components of the Pb displacements (a small isotropic component on top of the δ_Pb_) were allowed to swap (‘No swap δ_Pb_’), as was done with the SCDS modelling. The ‘Swap δ_Pb_’ case gave closer agreement with the PDF data compared with the ‘No swap δ_Pb_’ case, with fits of χ^2^ = 0.0003 and χ^2^ = 0.0021, respectively.

The SCDS calculated from these models is shown in Fig. 13 ▸. The diffuse rods of scattering are strongly present before the RMC. After the RMC simulation, in the case of allowing δ_Pb_ to swap (Fig. 13 ▸ *b*), the diffuse rods of scattering are almost completely gone, similar to the previous case where the planar domains were not induced (Fig. 12 ▸). When the δ_Pb_ were not allowed to swap (Fig. 13 ▸ *c*), the diffuse rods of scattering were forced to remain present. However, the other structured diffuse scattering that was formed was different from that observed in both Fig. 12 ▸(*b*) and in the data.

The correlations (Table 6 ▸) show that the RMC modelling increased the displacement correlations relative to the starting model for most of the different atom pairs, but the correlations remained weak. The strongest displacement correlation to form was between the O and *B*-site atoms for the case when δ_Pb_ were not allowed to swap and the planar domains were still present.

Whether forcing the model to contain polar nanodomains (PND) and *B*-site ordering or not, the displacement and occupancy correlations observed to form during the RMC analysis, although weak, were consistent in sign with those observed in the SCDS modelling and therefore provided guidance to what is happening on the nanoscale, especially if it is recognized that the real correlations are likely much stronger.

## 6. Conclusions   

The qualitative analysis of the PDF data in §2 showed that a Pb displacement direction of 〈110〉 is closest to what is observed in the data. There were also observed to be different separation distances between the two different *B*-site atoms and the neighbouring O atoms. This is information that is not contained in the average structure and can only be extracted from the SCDS through modelling. The PDF therefore immediately gives useful qualitative information that can feed into further modelling. This is important; the modelling of the SCDS relies relatively heavily on an investigator with wide experience of diffuse scattering and disorder, and if the PDF can deliver insights which *must* be incorporated into any valid model, and gives them relatively readily, this will aid less-experienced investigators in interpreting SCDS.

The PDF data allowed the evolution of the diffuse scattering to be observed with a higher temperature resolution because of the decrease in data collection time compared with SCDS. The derived ADPs were plotted against temperature and the greatest change was found to occur around the rhombohedral-to-cubic phase transition. The refined ADPs were found to be large, particularly for the Pb atoms, which is a sign that there is a large amount of disorder on that atom site and that a simple modelling approach cannot accurately model the nanostructure.

On calculating the PDF of the single-crystal model, it was observed that while the PDF contained much of the same information, it lacked the ability to distinguish some features that could be observed in the SCDS. The missing features included the *B*-site ordering [which produces strong diffuse peaks on the (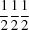)-type locations] and the formation of the planar nanodomains. The PDF data appear to be sensitive to the displacement of atoms, but less so to the correlation of these displacements. It is clear that the PDF contains information beyond the average structure that should be used to constrain a subsequent MC model, to help determine the ‘envelope’ within which the SCDS MC model *must* remain. Since the PDF (whether analysed by RMC or *PDFgui*) is clearly more quantitative than the SCDS when modelling the magnitudes of APDs, the way forward would seem to be a combination of the two approaches, possibly a form of joint refinement.

We note that if the material in question is not available as a single crystal, the results here still offer some useful ideas. When only PDF data are available it may be useful to calculate PDFs of a range of plausible local orderings using the experimental parameters (*Q*_max_ and so on) and compare them to gain some insight into the true discriminatory power of the experiment.

## Figures and Tables

**Table 1:** The fitted parameters, including ADPs, for the X-ray PDF data for the temperatures 100, 300 and 500 K, fitted using an average structure model in *PDFgui* The values of α and Pb_*x*,*y*,*z*_ were held fixed for the refinement.

Parameter	100 K	300 K	500 K
*a* (Å)	4.0595 (6)	4.0606 (7)	4.0618 (8)
α (°)	89.9	89.9	90.0
Pb_*x*,*y*,*z*_	0	0	0
Zn/Nb_*x*,*y*,*z*_	0.535 (1)	0.528 (2)	0.508 (8)
O_*x*_	0.009 (7)	0.003 (7)	-0.014 (1)
O_*y*,*z*_	0.580 (5)	0.577 (6)	0.563 (1)
*U*_Pb_	0.041 (2)	0.050 (2)	0.065 (3)
*U*_Zn/Nb_	0.006 (1)	0.009 (1)	0.013 (1)
*U*_O11_	0.093 (2)	0.087 (2)	0.081 (2)
*U*_O22_	0.055 (2)	0.059 (2)	0.071 (4)

**Table 2:** The fitted parameters, including ADPs, for the neutron PDF data for the temperatures 100, 300 and 500 K Note that here α is refined.

Parameter	100 K	300 K	500 K
*a* (Å)	4.0570 (1)	4.0585 (1)	4.0606 (1)
α (°)	89.8067 (1)	89.8247 (4)	90.0005 (3)
Pb_*x*,*y*,*z*_	0	0	0
Zn/Nb_*x*,*y*,*z*_	0.5277 (1)	0.5252 (1)	0.5382 (6)
O_*x*_	0.0406 (2)	0.0324 (1)	0.0388 (6)
O_*y*,*z*_	0.5537 (2)	0.5461 (1)	0.5441 (5)
*U*_Pb_	0.0397 (2)	0.0405 (1)	0.0213 (5)
*U*_Zn/Nb_	0.0071 (1)	0.0090 (1)	0.0108 (3)
*U*_O11_	0.0142 (2)	0.0139 (1)	0.0096 (5)
*U*_O22_	0.0189 (2)	0.0230 (1)	0.0216 (5)

**Table 3:** The parameters used for building the initial starting model crystal for use with RMC, based on the *PDFgui* fit to the neutron PDF at 300 K

Parameter	Value
*a* (Å)	4.0595
α (°)	89.865
Pb_*x*,*y*,*z*_	0
Zn/Nb_*x*,*y*,*z*_	0.5303
O_*x*_	0.0362
O_*y*,*z*_	0.5543
*B*_Pb_	3.1540
*B*_Zn/Nb_	0.8762
*B*_O_	1.6053

**Table 4:** The *B*-site occupancy correlations for two different neighbours, 〈100〉 and 〈110〉, for the rhombohedral model; all are very weak

Correlation	1.75 < *r* < 8 Å	1.75 < *r* < 20 Å
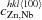	−0.085	−0.079
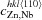	−0.004	0.070

**Table 5:** The displacement correlations for nearest-neighbour pairs in different symmetry directions Although they are weak correlations, those that are greater than 0.03 are highlighted in bold. The O1 atom considered as the origin for the calculation is at (0, 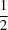, 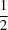).

Correlation	1.75 < *r* < 8 Å	1.75 < *r* < 20 Å
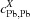	0.019	−0.020
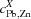	**0.033**	**0.032**
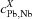	0.026	**0.042**
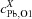	0.017	0.000
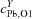	**−0.039**	−0.028
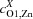	**0.054**	**0.109**
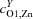	0.005	0.016
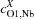	**0.081**	**0.156**
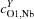	−0.002	0.023

**Table 6:** The displacement correlations for nearest-neighbour pairs in different symmetry directions for the planar domain model, where in one case the Pb atoms are allowed to swap while in the other case they are not Although they are weak correlations, those that are greater than 0.05 are highlighted in bold. The O1 atom that was used as the origin for the calculation is at (0, 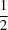, 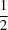)

	Starting model	Swap δ_Pb_	No swap δ_Pb_
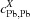	**0.1318**	0.0421	N/A
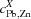	0.0059	**0.0508**	0.0245
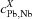	0.0019	0.0451	0.0486
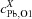	−0.0138	0.0029	−0.0001
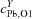	−0.0076	**−0.1066**	−0.0473
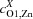	−0.0038	0.0593	**0.1526**
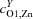	0.0152	0.0303	0.0146
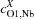	0.0043	**0.0799**	**0.1632**
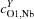	−0.0023	0.0261	0.0041
